# A spike-modified Middle East respiratory syndrome coronavirus (MERS-CoV) infectious clone elicits mild respiratory disease in infected rhesus macaques

**DOI:** 10.1038/s41598-018-28900-1

**Published:** 2018-07-16

**Authors:** Adam S. Cockrell, Joshua C. Johnson, Ian N. Moore, David X. Liu, Kevin W. Bock, Madeline G. Douglas, Rachel L. Graham, Jeffrey Solomon, Lisa Torzewski, Christopher Bartos, Randy Hart, Ralph S. Baric, Reed F. Johnson

**Affiliations:** 1 Department of Epidemiology, University of North Carolina-Chapel Hill, Chapel Hill, North Carolina, 27599 USA; 2 Integrated Research Facility, National Institute of Allergy and Infectious Diseases, National Institutes of Health, 8200 Research Plaza, Frederick, Maryland 21702 USA; 3 Infectious Disease Pathogenesis Section, Comparative Medicine Branch, Division of Intramural Research, National Institute of Allergy and Infectious Diseases, National Institutes of Health, Bethesda, MD 20892 USA; 4 Clinical Research Directorate/Clinical Monitoring Research Program, Leidos Biomedical Research, Inc., Frederick National Laboratory for Cancer Research, Frederick, Maryland 21702 USA; 5 Emerging Viral Pathogens Section, Laboratory of Immunoregulation, Division of Intramural Research, National Institute of Allergy and Infectious Diseases, National Institutes of Health, 8200 Research Plaza, Frederick, Maryland 21702 USA

## Abstract

The recurrence of new human cases of Middle East respiratory syndrome coronavirus (MERS-CoV) underscores the need for effective therapeutic countermeasures. Nonhuman primate models are considered the gold standard for preclinical evaluation of therapeutic countermeasures. However, MERS-CoV-induced severe respiratory disease in humans is associated with high viral loads in the lower respiratory tract, which may be difficult to achieve in nonhuman primate models. Considering this limitation, we wanted to ascertain the effectiveness of using a MERS-CoV infectious clone (icMERS-0) previously shown to replicate to higher titers than the wild-type EMC 2012 strain. We observed respiratory disease resulting from exposure to the icMERS-0 strain as measured by CT in rhesus monkeys with concomitant detection of virus antigen by immunohistochemistry. Overall, respiratory disease was mild and transient, resolving by day 30 post-infection. Although pulmonary disease was mild, these results demonstrate for the first time the utility of CT imaging to measure disease elicited by a MERS-CoV infectious clone system in nonhuman primate models.

## Introduction

Emerging in 2012, Middle East respiratory syndrome coronavirus (MERS-CoV) continues to be a threat to global human health. Twenty-seven countries have reported human cases of MERS-CoV, with all cases occurring in individuals returning from travel to the Arabian Peninsula or having contact with someone returning from the Arabian Peninsula (http://www.who.int/emergencies/mers-cov/en/). In May 2015, a single individual returning from travel to the Arabian Peninsula initiated an epidemic in South Korea that infected 186 people, and resulted in ~20% mortality^1^. Since January 2017, countries within the Arabian Peninsula reported monthly cases of MERS-CoV originating from either human contact with an infected individual or contact with dromedary camels, considered an intermediate zoonotic host (http://www.cidrap.umn.edu/). Infected individuals typically present with respiratory symptoms that can progress to severe pulmonary disease that is associated with a mortality rate of ~35% of reported cases^1^. Despite the threat to global public health, prevention of human-to-human transmission continues to rely upon standard public healthcare practices, and oxygen supplementation and mechanical respiratory support for infected individuals experiencing severe pulmonary complications^1^. Anti-MERS therapeutic and vaccine countermeasures are gradually advancing toward the clinic (reviewed in^1^); however, effective evaluation of anti-MERS countermeasures has been hampered, until recently, by the availability of animal models that reflect severe pulmonary complications observed during human infections^2,3^.

The approximate incubation period of MERS is 5 days (range 2–14 days), patients presenting with overt disease develop a fever, cough and shortness of breath. Severe pulmonary disease in humans has been associated with the detection of high viral loads in tracheal aspirates, sputum, throat swab, and blood or serum^4,5^. Recovery from severe respiratory disease may be associated with antibody and T cell responses^6^. The only publicly available autopsy report indicates lower respiratory tract (LRT) infection and a resulting pneumonia^7,8^. X-ray radiography and CT of patients also indicates a LRT disease in which pneumonia is the prominent finding (reviewed in^1^). Viral loads are highest in tracheal aspirates, which may contain increased material from the LRT based on ciliary motion. Therefore, an animal model that achieves pneumonia, and high viral loads in the trachea and lower respiratory tract (LRT) are important aspects to fully recapitulate severe human disease.

Three different research groups established models for MERS-CoV infection in two non-human primates (NHPs), the rhesus macaque and common marmoset^9–15^. Infection of the LRT could be established using different invasive strategies for placement of the challenge material^9,11,15^. The range of clinical outcomes included mild respiratory disease detectable using computed tomography and radiographic imaging^9,11,15^, to severe respiratory disease producing clinical endpoints indicative of a fatal disease that required euthanasia in two common marmoset models^9,15^. Two groups observed lethal disease following intratracheal, intranasal, and ocular exposure with the MERS-EMC isolate. However, Chan *et al*. indicate that lethality could be due to manipulations of marmosets, which are less hardy than macaque species^9^. A third group observed mild to moderate disease, but used higher viral titer, the MERS-Jordan isolate and a single route of challenge^11^. Experiments in which rhesus monkeys were used indicated that rhesus develop transient disease that peaks between day 3 and 5 post-infection and clears by day 14 post-infection^10^, however, gross and histopathology was not collected at peak of disease. Viral load peaks in this period in both marmosets and rhesus, and models in which subjects are euthanized at these early time points in disease progression have been used to demonstrate antiviral efficacy by measuring differences in viral load^16,17^. Overall, these disparate clinical outcomes indicate that the human strains of MERS-CoV used in the studies (EMC 2012 and Jordan) may not be suitably adapted to NHPs, thereby making it difficult for routine establishment of high viral loads in the LRT.

Understanding that it may be difficult to establish infection in NHPs with wild-type human MERS-CoV strains, we investigated the use of a novel MERS-CoV infectious clone. In previous studies, the MERS-0 infectious clone (icMERS-0) exhibited enhanced replication kinetics and higher titers than an EMC 2012 infectious clone on monkey kidney cells, and replicated to 10-fold higher titers than EMC 2012 in the lungs of infected mice^2^. The enhanced replication profile made icMERS-0 an attractive option for establishing infection in the rhesus macaque model. Intratracheal exposure of icMERS-0 elicited radiological and pathological signs of mild respiratory disease in the rhesus macaque. This is the first study demonstrating the capacity of a MERS-CoV infectious clone to elicit signs of respiratory disease in a NHP model. The work presented here expands the utility of the MERS-CoV reverse genetics systems for use in NHP models.

## Results

**Figure 1:**
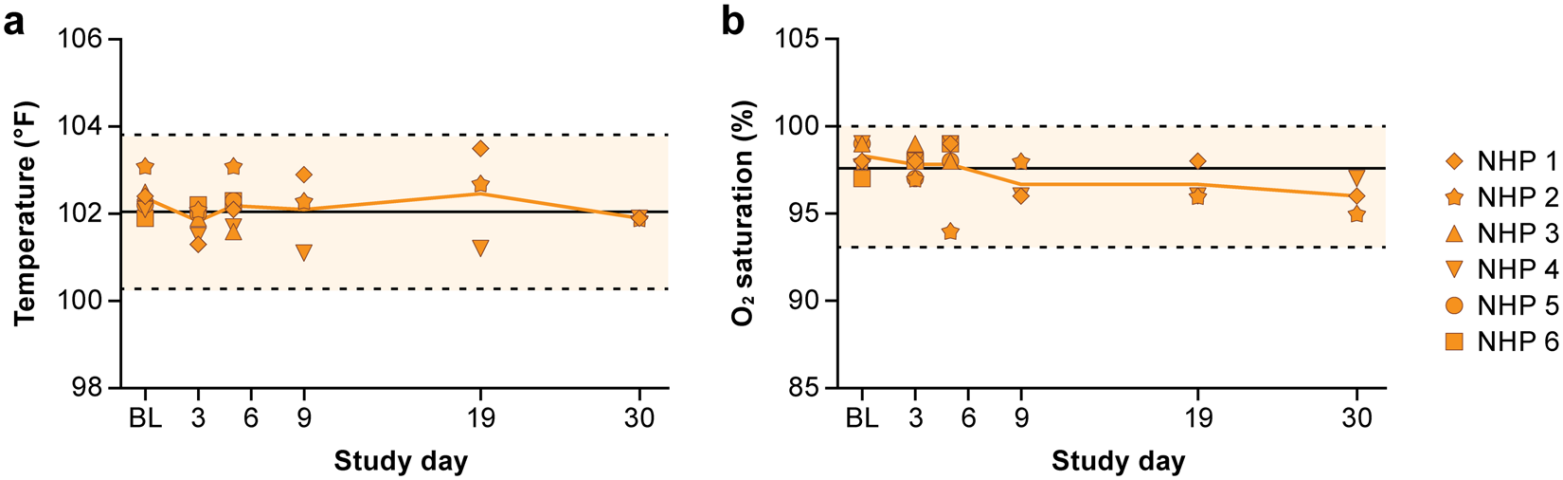
Temperature and arterial O2 saturation. (**a**) Study animals did not develop fever or deviate outside of normal macaque temperature ranges. (**b**) Animals did not exhibit O_2_ saturation loss outside of normal macaque ranges. Normal ranges are depicted in beige.

The icMERS-0 virus originates from the EMC 2012 genomic sequence and contains an additional insertion of three amino acids followed by a single amino acid change (S885L) in the S2 region of the spike gene, which augment replication^2^. The icMERS-0 virus was evaluated in six rhesus macaques that were survivors from a prior Ebola countermeasure efficacy experiment (Supplemental Fig. 1 for individual information). All six rhesus monkeys were infected with a target dose of 5 × 10^6^ PFU of icMERS-0 by intratracheal inoculation, with the entire dose of 6.62log_10_ PFU/mL placed at the carina. Rhesus monkeys were randomly assigned to two separate groups: an early time point group (day 5 post-infection) to examine pathology at peak disease (NHP3, NHP5, & NHP6) and a late time point group (day 30 post-infection) to monitor disease progression (NHP1, NHP2, & NHP4). All clinical signs (O_2_ saturation and temperature) remained within normal range throughout the entire experiment (Fig. 1). In accordance with clinical signs, complete blood counts with differential and serum chemistry analytes did not exhibit clinically significant changes from animal baselines (data not shown).

### Computed Tomography indicates that MERS-0 elicits mild respiratory disease

**Figure 2:**
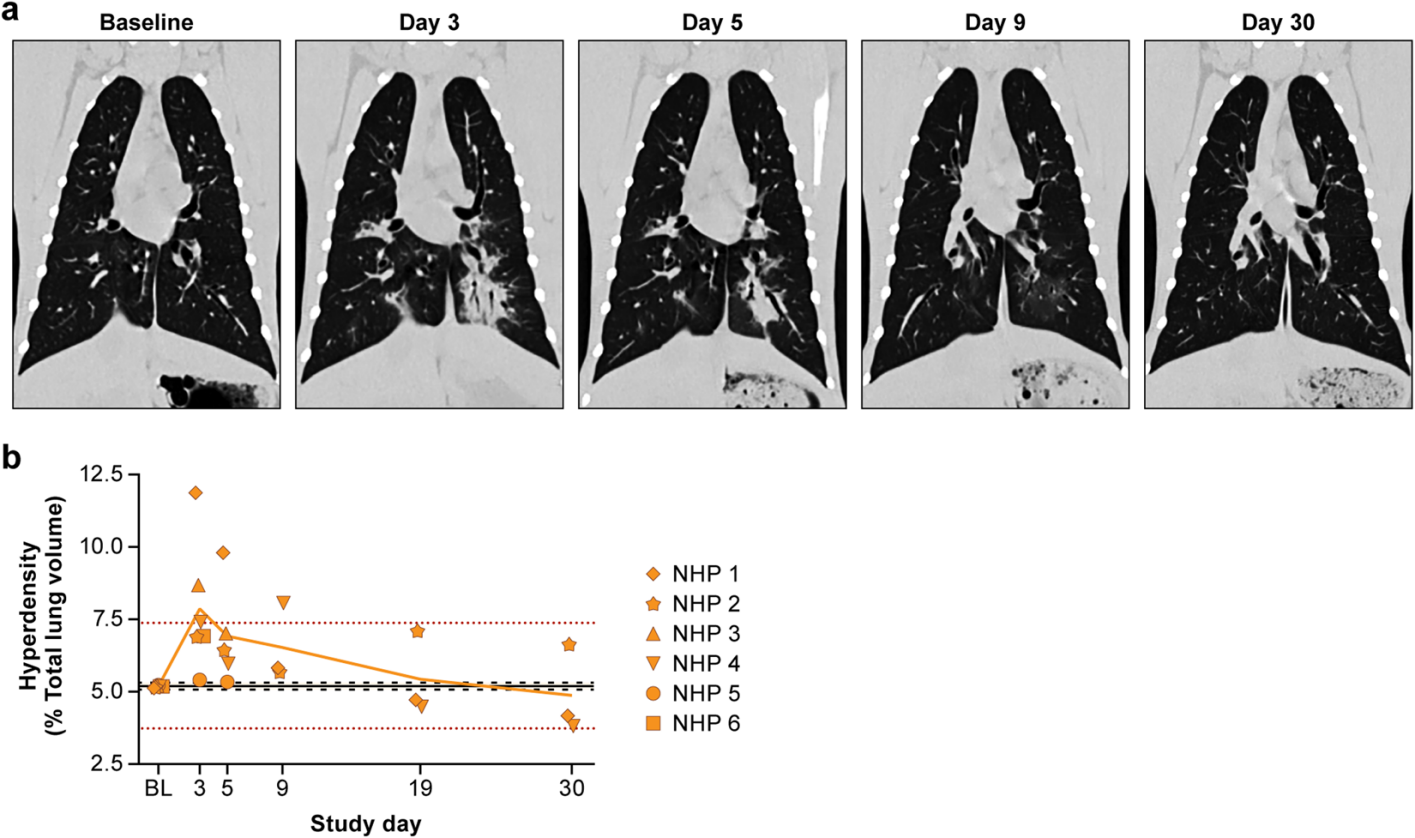
Percent change in lung hyperdensity (PCLH). (**a**) Representative lung field (**b**) Lung consolidation depicted as the percentage of the volume of hyperdense voxels in the total lung volume for each icMERS virus exposed animal. ^*^Day 5 for NHP6 was omitted due to improper endotracheal tube intubation for the imaging breadth hold. Solid and dotted black lines indicate the baseline mean and 3 SD range of the hyperdense volume from ic-MERS-0 exposed animals included in this study. Dotted red lines indicate the upper and lower method range limits of MERS exposed rhesus monkeys (n = 33).

Computed tomography (CT) imaging is a highly sensitive technique for evaluating tissues for pathological alterations. Pulmonary pathological changes can be quantified by measuring the intensity of hyperdense voxels in the total lung volume over the course of disease, also referred to as percent change in lung hyperdensity (PCLH)^18^. Areas of hyperdensity represent changes in lung tissue associated with lung pathology. NHP1 exhibited obvious increases in PCLH at days 3, with gradual resolution through day 30 post-infection (Fig. 2a). With the exception of NHP5, increases in PCLH were readily detectable over baseline in all NHPs over the course of disease (Fig. 2b). NHP5 exhibited no change in PCLH through the course of the experiment. NHP1, NHP3, NHP4, and NHP6 experienced increases that were above what was observed in prior NHP infections with wild-type MERS-CoV strains. It must be noted that improper endotracheal tube placement in NHP6 artificially exaggerated lung disease during the day 5 imaging session. Quantitative measurement of hyperdense regions indicate a peak at day 3 post-infection (p.i.) for NHPs that developed multifocal lung pathology which gradually resolved through day 30 p.i. (Fig. 2b). Nevertheless, CT demonstrates that icMERS-0 exposure resulted in quantifiable increases in lung pathology that were more severe than that previously observed with wild-type human strains^10,11^. Notably, disease in rhesus monkeys constitutes a mild respiratory infection compared to PCLH recalculated from data obtained from a previous study following infection with EMC 2012^11^ (Supplemental Fig. 2).

### Transient, mild pulmonary pathology in rhesus monkeys after MERS-0 inoculation

**Figure 3:**
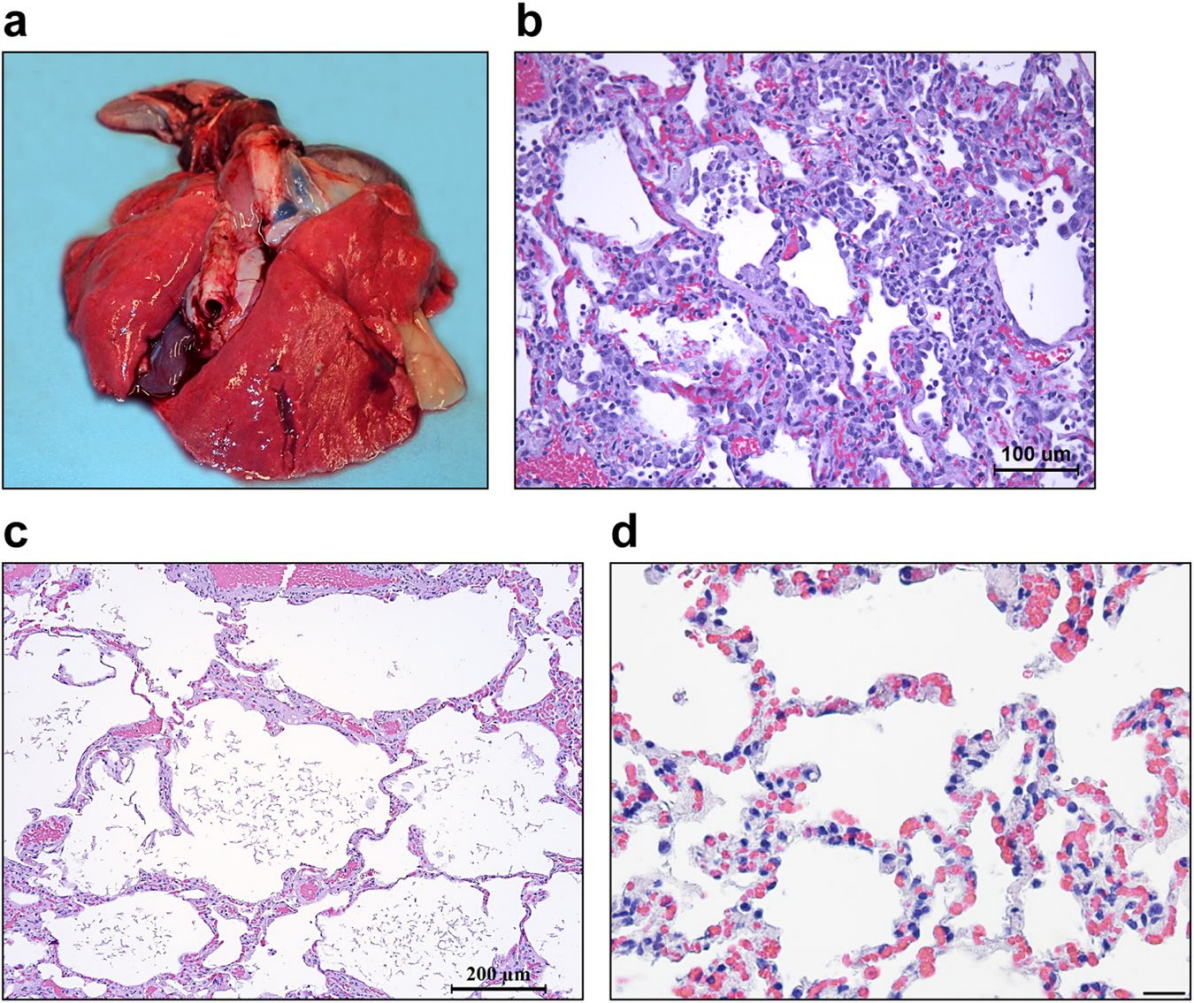
Pulmonary pathology in rhesus macaques inoculated with MERS-0. (**a**) Diffuse, mild pulmonary congestion. NHP3 at day 5 of MERS post-inoculation. (**b**) Multifocal, mild interstitial pneumonia characterized by type II pneumocyte hyperplasia and alveolar edema, fibrin and hemorrhage. NHP6 at day 5 of MERS post-inoculation. HE; (**c**) Multifocal alveolar emphysema containing small amount of eosinophilic to basophilic fibrillary material. NHP6 at day 5 of MERS post-inoculation. HE. (**d**) Day 30 H&E of lung demonstrating clearance of proteinaceous material and resolution of mild interstitial pneumonia.

Grossly, all 3 animals (NHP3, 5 and 6) euthanized at day 5 post-inoculation (pi) had diffuse, mild pulmonary congestion (Fig. 3a). Histologically, these 3 animals had focal-to-diffuse, minimal-to-mild interstitial pneumonia. The alveoli were lined by hyperplastic type II pneumocytes and filled with fibrin, edema, red blood cells, and macrophages. The septa were minimally-to-mildly expanded by fibrin, proteinaceous fluid, and mononuclear cells (Fig. 3b). Multifocally, other alveoli were often expanded and filled with eosinophilic, fibrillar material. The septa showed minimal to mild smooth muscle cell hyperplasia, fibrosis and vascular congestion (Fig. 3c). The three animals (NHP1, 2, and 4) necropsied on day 30 p.i. showed no significant lung lesions (Fig. 3d). Quantitation of microscopic findings in the lung are commensurate with histopathological findings (Supplemental Table 1), while extrapulmonary tissues exhibited Incidental histological changes (Supplemental Tables 2 and 3).

### Immunohistochemistry of MERS spike protein in the lungs

**Figure 4:**
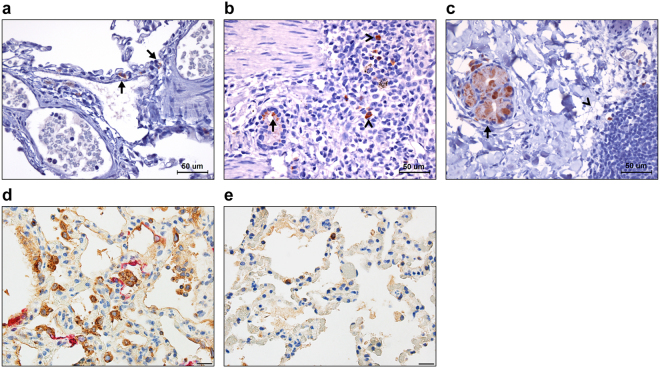
Immunohistochemistry of MERS spike protein in the lung. (**a**) Rare MERS spike antigen positive pneumocytes (arrows) of NHP6 at day 5 pi. (**b**) Rare MERS spike antigen positive cells in the submucosal glands (arrow) and lymphoid aggregates (^) of NHP6 at day 5 pi; (**c**) Many epithelial cells (arrow) of submucosal glands in the bronchi and fewer cells in the BALTs (^) were positive for MERS spike antigen of NHP2 at day 30 pi. (**d**) Increased numbers of alveolar macrophages that are positive for CD26 and MERS-CoV on day 5 pi. (**e**) Reduced hyperplasia and fewer CD26+ cells and alveolar macrophages were present on day 30 pi.

A scarce number of pneumocytes, perivascular inflammatory cells (Fig. 4a), epithelial cells of submucosal glands, and lymphoid aggregates (Fig. 4b) were positive for MERS spike protein in all 3 animals (NHP3, 5 and 6) euthanized at day 5 pi. In the lungs of 3 animals (NHP1, 2 and 4) at day 30 pi, more epithelial cells of submucosal glands in the bronchi and few cells in the BALTs were positive for MERS spike antigen (Fig. 4c). Dual immunohistochemistry staining for MERS spike and CD26 demonstrated MERS positivity in CD26 positive cells for all 3 subjects on day 5 pi and all three subjects were negative at 30 days post-infection (Fig. 4d,e). Relatively, alveolar macrophages were more prominent on day 5 pi than at day 30 pi. In contrast, epithelial cells of submucosal glands had more icMERS-0 antigen positive cells in 30 dpi group than the 5 dpi group. The small number of antigen positive cells increases the opportunity for error during lower lung aspirate sampling for RNA analysis, and may account for our inability to detect MERS viral RNA by qRT-PCR in lung aspirates (Supplemental Table 4). By focusing the RNA isolation on samples previously identified to be positive by IHC, icMERS-0 was detected in formalin-fixed, paraffin-embedded (FFPE) lung tissues (Table 1). The leader-ORF1 primer set detects full-length genomic and subgenomic RNAs, whereas the leader-ORFN combination detects subgenomic, replicating RNAs in infected cells. Subgenomic RNAs were detected in the 5 dpi group, but not in the 30 dpi group, indicative of higher levels of infection at 5 dpi (Table 1). Nonetheless, detection of icMERS-0 RNA in the 30 dpi group may be indicative of a viral adaptation and possible replication, but further studies would be required to confirm this hypothesis.

**Table 1:** icMERS-0 RNA from FFPE lung tissue.

Sample	Day PE	Lung Sample C_t_ Values		
		ORF1	ORFN	18S
DFLD – Lung R. Caudal	30	35.72	N	16.44
DFLD – Lung L. Cranial	30	N	N	16.22
DFBGA – Lung R. Caudal	5	37.02	37.22*	17.26
DFBGA – Lung L. Cranial	5	36.29	N	17.45
O5M – Lung R. Caudal	30	37.40	N	15.96
O5M – Lung L. Cranial	30	40.00	N	16.33
PH1071 – Lung R. Caudal	5	36.69*	N	17.78
PH1071 – Lung L. Cranial	5	36.75	34.17	17.52
DFCX – Lung R. Caudal	30	39.24	N	15.82
DFCX – Lung L. Caudal	30	N	N	16.05
A6V091 – Lung R Caudal	5	36.10	N	17.75
A6V091 – Lung L Caudal	5	32.24	31.19	17.44
A6V091 – Negative Control (Lung Aspirate)	5	N	N	10.32
A6V091 – Negative Control (Lung Aspirate)	5	N	N	10.71

C_t_ values represent an average of duplicate samples.

N is none detected.

*Represents a single value obtained for duplicate samples.

## Discussion

In this experiment, we have demonstrated that the icMERS-0 infectious clone derived from MERS-CoV is more pathogenic than the wild-type MERS-CoV Jordan isolate used in previous rhesus experiments. We have also demonstrated that disease elicited by icMERS-0 infection of rhesus peaked at D3–D5 post-exposure and resolves by D30 post-exposure. Infection corresponded with enhanced numbers of alveolar macrophages identified at D5 post-exposure, relative to day 30 post-exposure; similar to data previously reported for the rhesus macaque model^13,14^. The data further supports that the available MERS-CoV NHP models do not recapitulate human disease and that adaptation of MERS-CoV to NHPs may provide a suitable NHP model.

In the absence of an NHP model that reflects severe pulmonary disease with measurable clinical endpoints, having a noninvasive method for quantifying MERS-CoV-induced lung disease is important. Previous studies from our group demonstrate that the kinetics of MERS-CoV-induced mild lung disease can be readily measured using CT imaging combined with quantitation of the PCLH^10^. A previous study with MERS-CoV Jordan strain in the rhesus model established a range of values for PCLH analysis^10^. In the current study, icMERS-0 strain exhibited an increase in PCLH in 50% of the animals compared to that obtained with the Jordan strain from the prior rhesus study. Enhanced PCLH indicates that icMERS-0 may exhibit augmented pathogenesis compared to wild-type strains in the rhesus model; albeit additional studies directly comparing icMERS-0 to wild-type strains would be beneficial. Despite enhanced pathogenesis with icMERS-0, disease can be considered mild compared to the PCLH levels achieved in a rhesus model for smallpox utilizing cowpox virus^19,20^. PCLH levels are 10–80 fold higher in the rhesus smallpox model and are commensurate with overt signs of severe respiratory disease and mortality. These disparate outcomes in pulmonary disease severity indicate that MERS-CoV may not be adapted to overcome host immune mechanisms that preclude MERS-CoV pathogenesis in the rhesus model. Two approaches may facilitate development of models that reproducibly recapitulate human MERS-CoV pathogenesis: (i) alter the NHP host to one that is more susceptible to MERS-CoV infection and pathogenesis; or, (ii) adapt MERS-CoV in the rhesus model.

The common marmoset may be a more tractable NHP model for investigating MERS-CoV. More severe respiratory disease in the marmoset model, compared to the rhesus model, is reproducibly observed among all groups that have developed both pathogenic NHP models for MERS-CoV^9,11,15^. Two groups reported severe respiratory disease in the marmoset with clinical endpoint criteria indicative of a lethal disease requiring euthanasia^9,15^. In a third study the Jordan and EMC 2012 wild-type MERS-CoV strains exhibited large increases in the volume of lung tissue with pathological change, measured from CT images^11^. Direct quantitative comparisons of lesion volumes between rhesus and marmosets is complicated by differences in the animal lung volumes and the rapid breathing rate of marmosets compared to rhesus. Nonetheless, we were able to utilize data previously obtained following infection of marmosets with EMC 2012 to calculate the PCLH levels in a manner similar to that obtained for the rhesus monkeys (Supplemental Fig. 2). The higher PCLH levels in the marmosets indicate that more effective disease can be achieved in the marmosets. Accordingly, we propose that the icMERS-0 strain may elicit more severe respiratory disease in the marmoset model than that observed in the current study.

Technical differences that facilitate effective delivery of virus in the marmoset model, host genetic variation in the marmoset that facilitate infection, or host genetic variation in the rhesus macaque that curb infection may all contribute to differences in virulence observed between the two models. Since it is difficult to control for variation in host genetics in outbred NHP model systems, adapting MERS-CoV in the rhesus model may be a feasible option to circumvent these obstacles. The availability of animals and known reagents increase the feasibility and further enhance the attractiveness of the rhesus model. Regardless, proposed experiments should meet recommendations under the potential pandemic pathogen care and oversight (P3CO) policy guidance (https://www.phe.gov/s3/dualuse/Documents/P3CO-FinalGuidanceStatement.pdf).

The relative higher number of spike protein positive cells, and detection of icMERS-0 RNA, in epithelial cells of the submucosal gland at 30 dpi, in the absence of inflammatory cells, suggests the possibility that the icMERS-0 virus may have acquired mutations that facilitate long-term viral maintenance without immune detection. An increased number of MERS-CoV infected cells in the submucosa agrees with previous reports demonstrating robust expression of the DPP4 receptor in human submucosal gland epithelia compared to little expression in epithelia of the conducting airways^21,22^. Isolation of MERS-CoV from the submucosal glands could serve as a viral source for subsequent adaption in the rhesus macaque. Passaging of HIV-1 in the pigtailed macaque established precedent for adapting viruses in monkey models^23^. Only 4–5 passages of HIV-1 were required to achieve an NHP model that recapitulated AIDS observed in humans^23^. Further studies would be necessary to directly confirm persistence of low level MERS-CoV replication. Nonetheless, novel mutations identified by sequencing MERS-CoV RNA from the lungs could be incorporated into the MERS-CoV genome to generate novel infectious clones for subsequent passages that might yield an improved rhesus model. Moreover, a recombinant virus that produces disease in primates provides a strategy to evaluate virus genetic contributions to disease severity.

An NHP model that reproducibly recapitulates lethal human disease following respiratory infection may be effective for evaluating MERS-CoV countermeasures, but may be an oversimplification. A number of pre-existing conditions may contribute to an individual’s susceptibility to MERS-CoV. Increased age (>50) and several comorbidities (e.g. diabetes, hypertension, chronic respiratory disease, etc.) are commonly associated with individual’s that develop severe respiratory disease following MERS-CoV infection (reviewed in^1^). Capturing these complex phenotypes in a disease model may prove difficult and not cost effective. The combination of capturing complex phenotypes with the inability to control for host genetic variation in outbred NHP populations indicates that manipulating the host may prove difficult. Therefore, altering the MERS-CoV genome through adaptation may be the most feasible option for achieving a reproducible NHP model. The icMERS-0 strain demonstrates the feasibility of introducing novel genetic changes into the MERS-CoV genome that can augment respiratory disease phenotypes.

## Methods

### Methods to reduce study bias

Simple randomization was used to select the animals to be depopulated early at estimated peak disease to reduce the risk of decision bias on study outcomes. Randomization was performed using the RAND function in excel to assign a number between 0 and 1 to each NHP. The animals associated with the three highest results of the RAND function were selected for sacrifice on day 5.

### Virus challenge and dose quantitation

Preparation of virus inoculum and quantification of challenge dose MERS 0 (3T3p0, CCL-81p2) (MERS-0 in ref.^2^) was used for the challenge study. The ideal virus inoculum target was 6.7log_10_ PFU/mL to conform to historical studies. Virus inoculum was used undiluted as the initial virus stock titer was quantified at 6.6log_10_ PFU/mL average of two independent plaque assays on Vero cells (ATCC CCL-81).

Virus inoculum was back-titrated using the standard Avicel RC591® (FMC Biopolymer) semi-solid plaque assay method (CS-04–45). Briefly, virus inoculum was serially diluted by half-log increments in minimal essential medium alpha (Gibco) supplemented with Anti-Anti (Gibco) and heat-inactivated 5% fetal bovine serum (FBS, Gibco). Three-hundred microliters of sample was assayed on near-confluent 6-well plates of Vero cells (ATCC CCL-81) for 1 h at 37 °C and 5% CO_2_ with gentle agitation every 15 minutes to prevent drying of the monolayer. Following adsorption, 2 mL of a mixture of 2X eagle’s minimal essential medium (Gibco) and 2.5% Avicel RC591® was added to each well and plates were returned to the incubator for 3 d. Plate counts were enumerated after removing the overlay, staining with 0.2% gentian violet solution prepared in 10% neutral buffered formalin for 30 minutes, rinsing and drying the plates.

### Animal studies

Six rhesus monkeys, five of whom were previously survivors of an Ebola virus study evaluating efficacy of a polyclonal transgenic bovine antibody treatment, were used for this study 257 days post-Ebola virus infection. Animals were singly housed for the duration of the experiment and identified by both cage cards and tattoo markings. Animals were observed at least once daily following virus exposure. All animal experiments were performed in a BSL-4 laboratory, approved by the NIAID Division of Clinical Research Institutional Animal Care and Use Committee, and were performed in an AAALAC International accredited facility in accordance with relevant NIH policies and the Animal Welfare Act and Regulations.

Animals were anesthetized by immobilizing with a squeeze back cage to facilitate intramuscular delivery of ketamine prior to all animal handling, including physical exams and virus exposure. Virus exposure was performed by using a bronchoscope to introduce flexible tubing connected to a 3-way stop cock and deposit 0.5 mL of inoculum from a 3cc syringe into the left bronchus. The tubing was then flushed with 6cc of air using a 12cc syringe to ensure delivery of the entire volume. The process was repeated to introduce an additional 0.5 mL of inoculum to the right bronchus.

### Lung Computed Tomography Imaging and Quantitative Analysis

Animals were initially anesthetized using ketamine and anesthesia was maintained by intravenous propofol. Animals were intubated to facilitate breath hold for image clarity. Lung field CT images were acquired using a Philips Gemini CT scanner. Image analysis was performed using the percent change in lung hyper-density analysis (PCLH, Solomon *et al*. IEEE 27th International Symposium on Computer-Based Medical Systems) from which the hyperdensity volume as a percent of total lung volume was calculated by 100 × (hyperdense tissue volume/total lung volume). The analysis of the existing marmoset data set was performed in the same manner.

### Clinical sampling

Blood was collected in clot-activated tubes containing silica beads or K_3_EDTA anti-coagulant. Clot-activated tubes were allowed to clot for at least 30 minutes prior to processing. Blood tubes were centrifuged at 1800 relative-centrifugal force to separate serum or plasma upstream of chemistry analysis was performed using the Piccolo General Chemistry 13 discs and a Piccolo xPress analyzer or complete blood counts using the Sysmex XS-2000VT.

### Nasal swab and lung virology

Nasal swabs were collected using the BD Viral Transport system. Briefly, swabs were inserted into the nares and immediately transferred to the collection tubes containing virus stabilization medium using sterile technique. Swabs were vortexed vigorously for 30–60 seconds. Lung tissue samples were collected proximal to the bronchial branches, homogenized in bead beater tubes using an OmniRuptor and clarified by centrifugation prior to inactivation. Supernatants were inactivated in TRIzol LS in accordance with manufacturer’s instructions.

### RNA extraction and RT-qPCR

RNA from NHP lung aspirates was harvested in TRIzol and was isolated using the Zymo Direct-zol RNA MiniPrep Kit (Zymo Research Corp., Irvine, CA, USA) according to the manufacturer’s directions. RNA was isolated from formalin-fixed, paraffin-embedded lung tissues using Qiagen’s RNeasy Formalin-Fixed Paraffin-Embedded kit according to manufacturer’s instructions (Qiagen). First-strand cDNA was generated using Superscript III reverse transcriptase (Life Technologies, Carlsbad, CA, USA). For quantification of viral genome and subgenomic RNA, real-time PCR was performed using the SYBR-based SsoFast EvaGreen Supermix (Bio-Rad, Hercules, CA, USA) on a Roche LightCycler 480 (Roche, Basel, Switzerland). Real-time reactions used primer sets that specifically detected genome RNA (ORF1) and the nucleocapsid-encoding subgenomic RNA (ORFN) and are listed in supplemental 4. Results were then normalized to Eukaryotic 18 S and analyzed using the ΔΔCt method.

### Gross and Histological Pathology

All animals underwent an extensive postmortem necropsy by a qualified pathologist. The animal’s overall general condition and state of major organ systems was assessed. Tissues trimmed to 1 cubic centimeter, submerged and fixed in 10% neutral buffered formalin for 7 days. Paraffin embedded tissue sections were mounted on positively-charged slides and stained with hematoxylin and eosin prior to pathological examination.

### Immunohistochemistry

Immunohistochemistry targeting the MERS-CoV spike protein was performed on lung tissue samples. Tissue slides were subjected to heat-induced antigen retrieval with a citrate based buffer for 30 minutes at 95 °C (Biocare Medical, Cat # DV2004) and protein blocked (Biocare Medical, Cat #BS966MM). Immunostaining was performed using a primary rabbit polyclonal antibody specific for MERS-CoV spike protein (Sino Biological, Cat #40069-RP02) diluted 1:1000 followed by a secondary biotinylated goat anti-rabbit IgG (Jackson Immunoresearch, Cat #111-065-144) at 1:1500 dilution and resolved using the VECTASTAIN Elite ABC HRP kit (Vector Labs, Cat #PK-6100) and Betazoid DAB Kit (Biocare Medical, Cat #BDB2004L).

Sequential double staining was carried out on formalin-fixed paraffin-embedded lung sections using goat polyclonal anti-CD26 (R&D Systems, Cat #AF1180) and mouse monoclonal anti-MERS CoV nucleocapsid (NP) protein (Biorbyt LLC, Ca t #Q11974). Protocols were completed on the Bond RX (Leica Biosystems) platform. Briefly, 5µm-thick sections were deparaffinized and rehydrated. Heat-induced epitope retrieval (HIER) was performed using Epitope Retrieval Solution 1, pH 6.0, heated to 100 °C for 20 minutes. The specimen was then incubated with hydrogen peroxide to quench endogenous peroxidase activity prior to applying the anti-CD26 at 1:1000 for 15 minutes. Detection with DAB chromogen was completed using the Bond Polymer Refine Detection kit (Leica Biosystems, Cat #DS9800), with the Post Primary step being replace by an eight-minute incubation with rabbit anti-goat secondary antibody (Abcam, Cat #ab6740). Anti-MERS NP was then applied at 1:100 for 15 minutes. Fast Red detection was performed with the Bond Polymer Refine Red Detection kit (Leica Biosystems, Cat #DS9390), including counterstaining with hematoxylin. Slides were finally cleared through gradient alcohol and xylene washes prior to mounting and coverslipping. Sections were examined by a board-certified veterinary pathologist (INM) using an Olympus BX51 light microscope and photomicrographs were taken using an Olympus DP73 camera.

## Electronic supplementary material

Dataset 1
