# Supplementary material for: A spike-modified Middle East respiratory syndrome coronavirus (MERS-CoV) infectious clone elicits mild respiratory disease in infected rhesus macaques
Source: Sci Rep. 2018 Jul 16;8:10727. doi: 10.1038/s41598-018-28900-1 (PMC6048037; doi:10.1038/s41598-018-28900-1)
Supplement: Supplementary file 1 — Dataset 1 [file 41598_2018_28900_MOESM1_ESM.pdf]

Supplementary Information

A spike-modified Middle East respiratory syndrome coronavirus (MERS-CoV) infectious clone elicits mild respiratory disease in infected rhesus macaques.

Adam S. Cockrell*, Joshua C. Johnson, Ian N. Moore, David X. Liu, Kevin W. Bock, Madeline G. Douglas, Rachel L. Graham, Jeffrey Solomon, Lisa Torzewski, Christopher Bartos, Randy Hart, Ralph S. Baric*,#, Reed F. Johnson*,#

*Co-corresponding authors: Address Correspondance to: A.S.C. (adam_cockrell@unc.edu), R.S.B. (baric@email.unc.edu), or R.F.J. (johnsonreed@niaid.nih.gov)

#Co-senior authors: R.S.B and R.F.J.

Supplementary Tables 1-5

Supplementary Figures 1-2

Supplemental Table 1. Microscopic findings in the lung.

Lesions		NHP6	NHP3	NHP5	NHP2	NHP1	NHP4
		Day 5 Post-Exposure			Day 30 Post-Exposure		
Alveoli	Edema & fibrin	3 (fe)	2 (fe)	1 (mf)	0	3 (fe)	0
	Hemorrhage	3 (fe)	2 (fe)	2 (fe)	0	1 (fe)	0
	Emphysema[1]	2 (mf)	2 (mf)	2 (mf)	0	2 (mf)	2 (mf)
	Histiocytosis	1 (mf)	2 (fe)[2]	1 (mf)	0	2 (mf)[4]	1 (mf)
Septa	Type II pneumocyte hyperplasia	2 (fe)	2 (fe)	1 (mf)	0	0	0
	Smooth muscle hyperplasia	2 (mf)	2 (fe)	2 (mf)	1 (mf)	2 (fe)	3 (fe)
	Fibrosis	2 (mf)	2 (fe)	1 (mf)	0	2 (fe)	2 (fe)
	Infiltrates of lympho-plasmacytic-monocytic cells	2 (mf)	1 (mf)	1 (mf)	0	2 (fe)	2 (fe)
Blood vessels	Congestion	2 (mf)	2 (mf)	2 (mf)	0	1 (mf)	0
	Perivasculitis	2 (mf)	2 (mf)	1(mf)	0	1 (mf)	0
	BALT[3] hyperplasia	0	0	0	2 (mf)	2 (mf)	2 (mf)
Bronchi & bronchioles	Collapsed	2 (mf)	2 (mf)	1 (mf)	2 (mf)	1 (mf)[5]	2 (mf)
	Goblet cell hyperplasia	0	0	0	2 (mf)	2 (mf)	2 (mf)
Pleura	Lymphoplasmacytic and edematous	2 (df)	0	0	0	0	0

Abbreviations: fe, focally extensive; mf, multifocal; df, diffuse. Qualitative scoring scale: 0, within normal limits; 1, mild; 2, moderate; 3, significant; 4, severe. [1] Filled with eosinophilic to basophilic fibrillar material; [2] Many multinucleate giant cells and plant material; [3] BALT, bronchial associated lymphoid tissue; [4] Granulomas with nematodes; [5] Filled with large amount of mucinous material and cell debris.

Supplemental Table 2. Microscopic findings in the lymphoid organs.

Lesions		NHP6	NHP3	NHP5	NHP2	NHP1	NHP4
		Day 5 Post-Exposure			Day 30 Post-Exposure		
Axillary lymph node	Lymphoid depletion and hyperplasia	1 (df)	2 (df)	2 (df)	2 (mf)	0	0
	Lymphocytic hyperplasia	2 (df)	3 (df)	2 (df)	2 (df)	0	2 (df)
	Sinus dilatation	2 (df)	0	0	0	0	0
	Hystiocytosis	2 (mf)	2 (df)	2 (df)	2 (df)	2 (mf)	1 (mf)
Inguinal lymph node	Lymphoid depletion and hyperplasia	NA	2 (df)	2 (df)	0	0	0
	Lymphocytic hyperplasia		3 (df)	2 (df)	2 (df)	0	2 (fe)
	Sinus dilatation		0	0	0	0	0 (fe)
	Hystiocytosis		2 (mf)[1]	2 (df)	2 (df)	2 (fe)	1 (fe)
Mesenteric lymph node	Lymphoid depletion and hyperplasia	0	0	3 (mf)	3 (mf)	2 (mf)	1 (mf)
	Lymphocytic hyperplasia	0	0	0	0	0	0
	Sinus dilatation	2 (mf)	2 (mf)	0	0	0	0
	Hystiocytosis	2 (mf)	2 (mf)	2 (mf)[2]	2 (df)[3]	2 (mf)[2]	2 (mf)[2]
Bronchial lymph node	Lymphoid depletion and hyperplasia	NA	2 (mf)	0	3 (mf)	2 (mf)	1 (mf)
	Lymphocytic hyperplasia		2 (df)	2 (df)	0	0	0
	Sinus dilatation		0	0	0	0	0
	Hystiocytosis		0	0	0	0	0
Spleen	Lymphoid depletion and hyperplasia	2 (mf)	2 (df)	0	0	0	0
	Lymphocytic hyperplasia	2 (mf)	2 (df)	0	0	0	0
	Sinus dilatation	0	0	0	0	0	0
	Hystiocytosis	0	0	0	0	0	0

Abbreviations: fe, focally extensive; mf, multifocal; df, diffuse. Qualitative scoring scale: 0, within normal limits; 1, mild; 2, moderate; 3, significant; 4, severe. [1] with mild hemosiderionosis; [2] Granulomas with nematodes; [3] Granulomatous inflammation;

Supplemental Table 3. Incidental microscopic findings.

Organ / Lesions		NHP6	NHP3	NHP5	NHP2	NHP1	NHP4
		Day 5 Post-Exposure			Day 30 Post-Exposure		
Skin & mammary gland		0	0	0	0	0	0
Kidney	Lymphocytic interstitial nephritis	1 (mf)	0	0	0	0	1 (mf)
Heart	Lymphoplasmacytic perivasculitis	1 (mf)	0	0	1 (mf)	0	0
	Adipose tissue infiltration	2 (mf)	0	0	0	0	0
	Fibrosis	1 (mf)	0	0	1 (mf)	1 (mf)	0
Tongue		0	0	0	0	0	0
Liver	Lipidosis	2 (mf)	0	2 (mf)	0	0	0
	Lymphoplasmacytic	0	0	0	1 (mf)	0	0
Brain	Lymphoplasmacytic	0	0	0	1 (mf)	0	0
Pituitary gland		0	0	0	0	0	0

Abbreviations: mf, multifocal. Qualitative scoring scale: 0, within normal limits; 1, mild; 2, moderate; 3, significant; 4, severe.

Supplementary Table 4. Results of MERS-CoV real-time quantitative polymerase chain reaction. All real-time runs were 45 cycles. The positive control was an infected sample from a human primary lung culture.

Primate #	Sample Number	Day PE	Lung Sample Ct Values		
			ORF1	ORFN	18S
NHP1	1	30	N	N	16.88
	1	30	N	N	15.86
	2	30	N	N	14.99
	2	30	N	N	14.03
NHP2	1	30	N	N	13.37
	1	30	N	N	12.78
	2	30	N	N	12.73
	2	30	N	N	12.91
NHP3	1	5	N	N	13.34
	1	5	N	N	13.57
	2	5	N	N	15.20
	2	5	N	N	15.26
NHP4	1	30	N	N	11.81
	1	30	N	N	12.33
	2	30	N	N	13.16
	2	30	N	N	12.74
NHP5	1	5	N	N	16.52
	1	5	N	N	15.91

	2	5	N	N	16.77
	2	5	N	N	17.05
**NHP6**	1	5	N	N	20.57
	1	5	N	N	20.98
	2	5	N	N	17.05
	2	5	N	N	16.24
**Positive Control**	n/a	n/a	15.41	21.68333333	11.54

Abbreviations: PE, post-exposure; 18S, eukaryotic 18S; N, negative (no reads through 45 cycles).

Supplemental Table 5. Coronavirus genomic and subgenomic real-time primer sets and control primers.

Primer Name	Primer Sequence (5’-3’)
MERS Leader Forward	GAATAGCTTGGCTATCTCAC
MERS ORF1 Reverse	CACAATCCCACCAGACAA
MERS ORFN Reverse	TTGTTATCGGCAAAGGAAAC
Euk18S Forward*	AATTTGACTCAACACGGG
Euk18S Reverse*	ACTAAGAACGGCCATGCACC

* The eukaryotic housekeeping gene 18S (Euk18S) was used for normalization of real-time results.

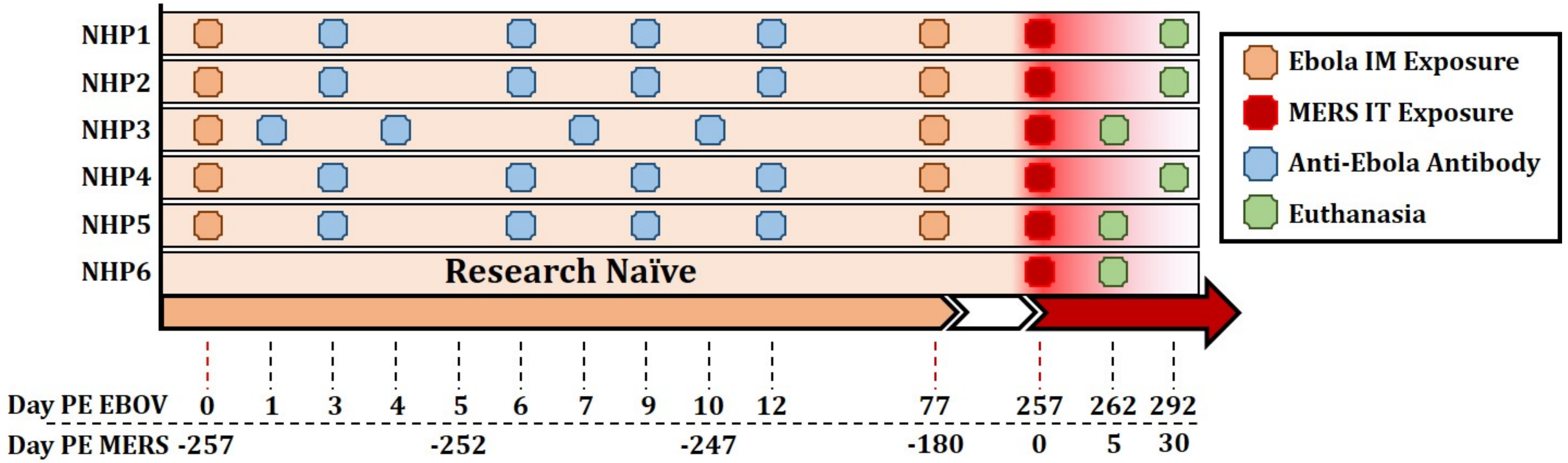

Supplemental Figure 1. Nonhuman primate research history. Five of the six nonhuman primates used for the performance of this study survived two previous intramuscular exposures to Ebola virus (2300 plaque-forming units and 611 plaque-forming units for the first and second Ebola exposure, respectively). Animals survived due to receiving intravenous infusions of anti-Ebola polyclonal antibodies generated from humanized transgenic cows.

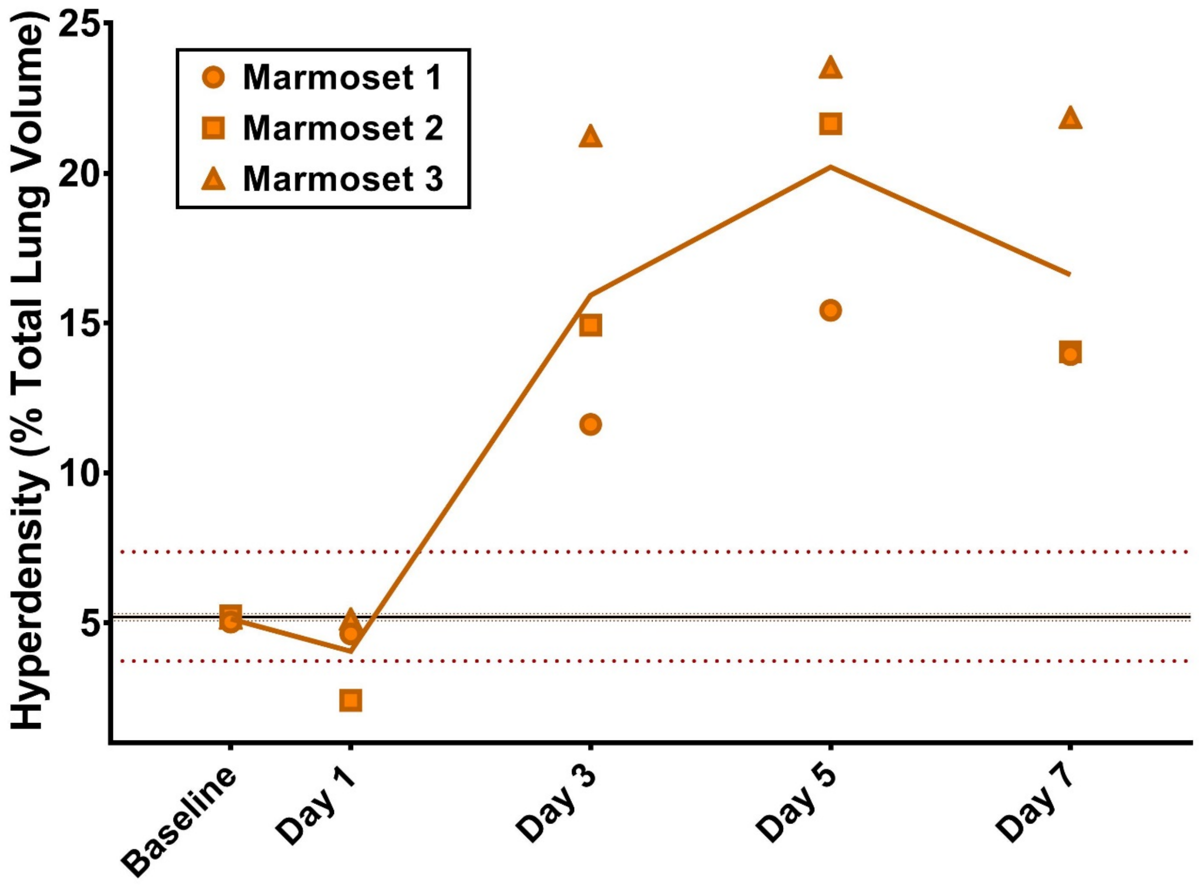

Supplemental Figure 2. (A) Representative lung field CT images of a marmoset 1 exposed to 5 x 10^7 PFU MERS EMC virus by the intratracheal route; (B) Lung consolidation depicted as the percentage of the volume of hyperdense voxels in the total lung volume for each MERS EMC virus exposed marmoset. Solid and dotted black lines indicate the baseline mean and 3SD range of the hyperdense volume from icMERS-0 exposed rhesus monkeys. Dotted red lines indicate the upper and lower method range limits of MERS exposed rhesus monkeys (n=33). Marmoset data used in this figure was originally reported in [7].
